# Predictors of Incident Benzodiazepine Co-prescription Among Patients Prescribed Long-term Opioids

**DOI:** 10.1007/s11606-025-09712-2

**Published:** 2025-07-16

**Authors:** Iraklis Erik Tseregounis, Stephen G. Henry, Shao-You Fang, Susan Stewart, Alicia Agnoli, James J. Gasper, Joshua J. Fenton

**Affiliations:** 1 Department of Internal Medicine, UC Davis, Sacramento, CA, USA; 2 Center for Healthcare Policy and Research, UC Davis, Sacramento, CA, USA; 3 Department of Public Health Sciences, UC Davis, Davis, CA, USA; 4 Department of Family and Community Medicine, UC Davis, Sacramento, CA, USA; 5 Department of Family and Community Medicine, UC San Francisco, San Francisco, CA, USA

**Keywords:** opioids, benzodiazepines, co-prescription, incident

## Abstract

**BACKGROUND:**

Opioid and benzodiazepine co-prescription is associated with overdose, particularly among patients prescribed long-term opioids.

**OBJECTIVES:**

Identify predictors of incident benzodiazepine and opioid co-prescription using two separate and complementary large-scale patient cohorts.

**DESIGN:**

Two retrospective cohort studies: (a) statewide dataset based on California’s prescription drug monitoring program (PDMP, 7/1/2016–12/1/2018) and (b) national sample of commercial and Medicare Advantage enrollees from the Optum Labs Data Warehouse (OLDW, 7/1/2016–12/1/2021).

**PARTICIPANTS:**

Patients prescribed long-term opioids, with opioid coverage for ≥ 80% (≥ 144 days) of a 180-day baseline period absent baseline benzodiazepine or buprenorphine prescriptions. OLDW cohort excluded patients without continuous enrollment, with cancer diagnoses or use of hospice or prolonged inpatient skilled nursing care.

**MAIN MEASURES:**

Incident benzodiazepine and opioid co-prescription (≥ 20 days of co-prescription during any 30-day period).

**KEY RESULTS:**

Of 617,946 and 223,885 patients, incidence rates of co-prescription were 4.6 and 3.9 cases per 1000 patient-months in the PDMP and OLDW cohorts, respectively. Important predictors included patients prescribed > 150 mg morphine equivalents daily during baseline (PDMP, adjusted hazard ratio: 1.74 [95% CI: 1.67–1.81]; OLDW: 2.66 [2.47–2.86]), and initiated buprenorphine indicated for treatment of opioid use disorder, with (PDMP: 1.68 [1.49–1.89]; OLDW: 2.10 [1.71–2.59]) or without continued treatment (PDMP: 1.35 [1.18–1.56]; OLDW: 1.64 [1.27–2.11]). Co-prescription was positively associated with short-term (60-day) decreases in opioid dose (PDMP: 1.07 [1.04–1.10]; OLDW: 1.06 [1.01–1.12]) but negatively associated with long-term (180-day) decreases (PDMP: 0.81 [0.78–0.85]; OLDW: 0.78 [0.73–0.84]). Patients with anxiety diagnoses were at elevated risk for co-prescription (OLDW: 2.16 [2.06–2.27]), although risk was lower if accompanied by treatment with serotonergic anxiolytics (0.63 [0.59–0.67]).

**CONCLUSIONS:**

High baseline opioid dose, buprenorphine initiation, short-term decrease in opioid dose, and anxiety without prescriptions for serotonergic anxiolytics were positively associated with co-prescription. A longer-term decrease in opioid dose and anxiety treated with serotonergic anxiolytics were negatively associated with co-prescription.

## INTRODUCTION

An increasing proportion of opioid overdoses in the USA also involve benzodiazepines.^1,2^ Opioids and benzodiazepines have synergistic effects on suppressing respiratory drive,^3^ and patients with concurrent use have a 2- to tenfold increased risk of overdose compared to patients prescribed opioids alone.^4–7^

Despite documented risks and longstanding recommendations to avoid the combination,^1,8^ benzodiazepine and opioid co-prescription remains common. Studies among commercially insured and Medicare Advantage patients, using the Optum Labs Data Warehouse (OLDW) commercial claims database, have found that 1 in 4 patients on long-term opioid therapy are co-prescribed benzodiazepines.^9,10^

While concurrent opioid and benzodiazepine use is a well-established overdose risk factor, factors associated with the transition to co-prescription are not well characterized.^11^ Benzodiazepines are often prescribed to treat comorbidities that are common in patients with chronic pain such as anxiety, muscle spasm, or insomnia.^12^ As with prescription opioids, patients who initiate benzodiazepine therapy may continue benzodiazepine use long-term, resulting in physical dependence, tolerance, and withdrawal symptoms that complicate efforts to reduce or stop the medication.^13^ Improved understanding of the risk factors for incident benzodiazepine and opioid co-prescription is crucial for developing prevention strategies to reduce overdose and related outcomes.

To characterize these risk factors, we conducted two parallel cohort studies of patients prescribed long-term opioids to identify predictors for incident benzodiazepine and opioid co-prescription. We separately analyzed two complementary datasets: statewide prescription records data from California’s prescription drug monitoring program (PDMP) and enrollment and claims from a national patient sample derived from the OLDW. Similar findings from these two cohorts would suggest that observed risk factors are robust and generalizable, and not merely due to geographic or administrative characteristics of either dataset alone.

## METHODS

### Ethics

This study was approved, with requirements for patient consent waived, by the California Committee for the Protection of Human Subjects and the University of California, Davis Institutional Review Board. The study protocol was pre-registered at AsPredicted.org (#133335).

### Data Sources

Two data sources were utilized to conduct complementary and independent analyses. The first data source comprised controlled substance prescription records from California’s PDMP dispensed between January 1, 2016, and December 31, 2018. California’s PDMP contains statewide data on all outpatient Schedule II-IV prescriptions dispensed by California outpatient pharmacies. Prescription records included patient, prescriber, and pharmacy information. Each prescription record also included date dispensed, National Drug Codes, quantity, strength per unit, and days’ supply. Prescription records were linked to California death certificate data using patient date of birth, sex, name, and address. Linked data were then de-identified for analysis. The second data source was the OLDW with claims records from January 1, 2016, through December 31, 2021. The OLDW contains de-identified retrospective administrative data, including medical and pharmacy claims (with associated diagnosis codes) and eligibility information for commercial and Medicare Advantage enrollees. The database contains longitudinal health information on patients representing a mix of ages and US geographical regions.^14^

These two data sources have complementary strengths. The California PDMP captures nearly all outpatient controlled substance prescriptions in California statewide (12% the US population) regardless of insurance status. The OLDW is a national sample of patients with commercial insurance or Medicare Advantage with linked clinical claims in a sample representing over 20% of the commercial and Medicare Advantage markets nationally.

### Cohort Identification

**Figure 1:**
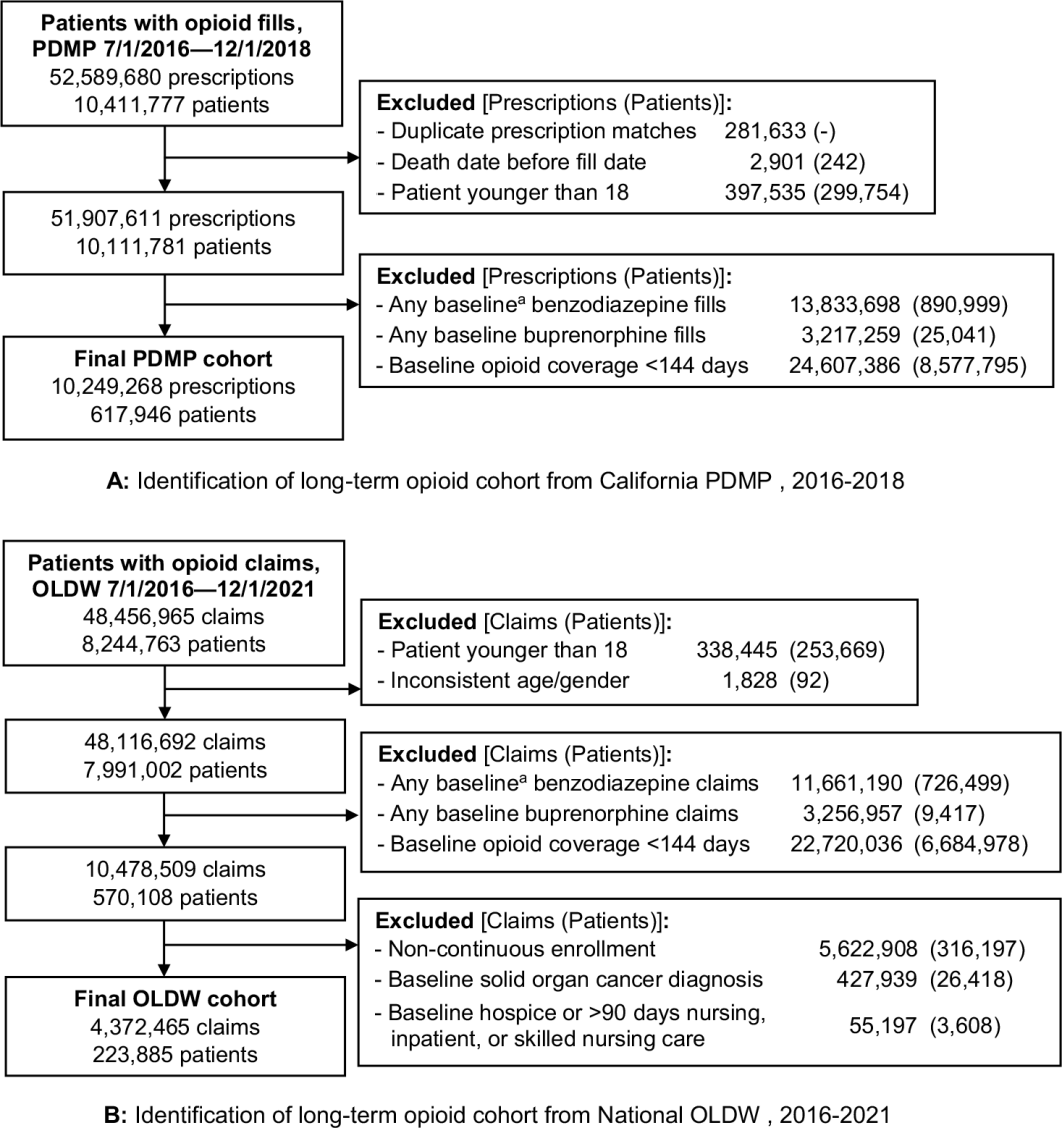
A Identification of long-term opioid cohort from California PDMP, 2016–2018. B Identification of long-term opioid cohort from National OLDW, 2016–2021. PDMP, California prescription drug monitoring program data; OLDW, Optum Labs Data Warehouse. ^a^180-day period prior to study entry.

Separate cohorts were derived from each dataset (Fig. 1A/B), and included patients aged ≥ 18 years with at least one opioid prescription fill between July 1, 2016, and December 1, 2018 for the PDMP cohort, and between July 1, 2016, and December 1, 2021, for the OLDW cohort. Patients were eligible to enter the cohort if, on the first day of any given opioid fill, they had opioid coverage for at least 144 days (80%) of the prior 180 days (baseline period). Patients were excluded if they had any benzodiazepine or buprenorphine prescription, indicated for the treatment of opioid use disorder, during this 180-day baseline period. Patients with buprenorphine coverage during baseline were excluded to ensure that the sample comprised patients receiving opioids for chronic pain rather than opioid use disorder, though we did not exclude patients who received buprenorphine during the follow-up period.

The OLDW cohort dataset had additional cohort eligibility criteria. Only patients continuously enrolled in medical, pharmacy, and mental health coverage for 365 days prior to their index opioid fill date and for at least 30 days after their index fill date were eligible for inclusion. Additionally, we excluded patients with solid organ cancer diagnoses (International Classification of Diseases, Clinical Modification, Tenth Revision [ICD-10-CM] codes C00–C41, C43, C45–C96, D00–D09, D47.29, D46.9, E31.22, E31.23), any claims for hospice/palliative care, or ≥ 90 days of nursing, inpatient, or skilled nursing care during the 365-day period prior to the index opioid fill date.

### Study Design

**Figure 2:**
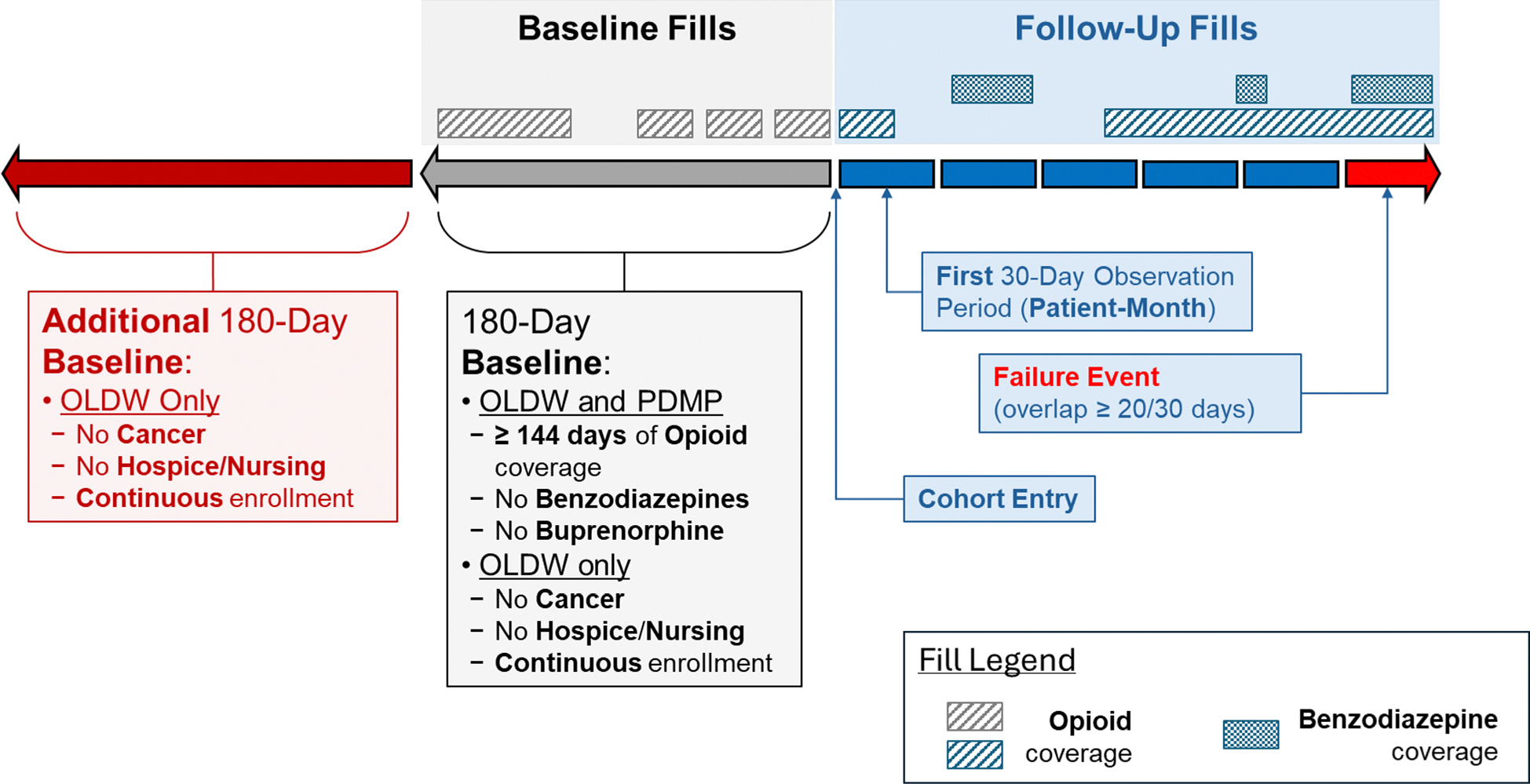
Patient follow-up timeline and example. OLDW, Optum Labs Data Warehouse; PDMP, prescription drug monitoring program.

For both datasets, upon cohort entry, patients were followed for the duration of the study period (PDMP study end: December 31, 2018; OLDW study end: December 31, 2021) until they met the definition for co-prescription or were censored due to patient death. Exclusive to the OLDW cohort, additional censoring events included health plan disenrollment, new solid tumor cancer diagnoses, initiation of hospice or palliative care, or receipt of ≥ 90 days of skilled nursing care. Follow-up was categorized into discrete 30-day (patient-month) observation periods starting on the date of each patient’s cohort entry. An example patient timeline is presented in Fig. 2.

### Dependent Variable

The study outcome was incident benzodiazepine and opioid co-prescription, defined as the occurrence of ≥ 20-day supply of overlapping opioid and benzodiazepine prescriptions during any patient-month in the study period. As this definition is a shorter assessment window than the 90-day periods used in other studies,^15,16^ we also conducted sensitivity analyses using a ≥ 60-day supply of overlapping opioid and benzodiazepine coverage during any 3 patient-months (90 days total) in the study period.

### Independent Variables

Time invariant independent variables common to both cohorts included patient demographics (age, sex), calendar year of cohort entry, and opioid prescribing patterns during the baseline period (180-day average of daily milligram morphine equivalents [MME], predominant opioid type, number of unique opioid prescribers).

Time-varying independent variables common to both cohorts included prior 180-day opioid prescribing characteristics (any long-acting formulation, any overlap of opioid prescriptions), prior 180-day additional controlled substance prescribing (any opioid and non-benzodiazepine sedative [including controlled sedatives such as zolpidem, zaleplon, eszopiclone, and carisoprodol in both cohorts, and also gabapentinoids in the OLDW cohort] prescription overlaps, any active psychostimulant prescription), and buprenorphine, indicated for the treatment of opioid use disorder, prescriptions during follow-up (none, initiation during follow-up and buprenorphine prescription in the prior 30 days, initiation during follow-up but no active prescription in the prior 30 days).

Prior work identified that opioid dose changes may be associated with adverse mental health outcomes^10,17^ and therefore potential subsequent benzodiazepine use. To examine whether patients’ opioid dose trajectory was associated with incident co-prescription, we included changes in opioid dose, MME trajectory, in the preceding 60-day (“short term”) or 180-day (“long-term”) prescribing period, long-term opioid dose variability,^18^ and opioid discontinuation status. See Appendix 1 of the supplement for details on opioid trajectory variable construction and definitions.

Time invariant independent variables exclusive to the OLDW cohort included commercial vs. Medicare Advantage patient insurance status and area-level (Census block) demographics (rurality, median education level, median household income), patient history of anxiety, depression, psychoses, alcohol and substance use disorders using ICD-10-CM diagnosis codes, Elixhauser comorbidity indicators,^19^ and any overdose resulting in an emergency department or hospital visit occurring in the 365-day period prior to study entry.

Time-varying independent variables exclusive to the OLDW cohort included past 180-day prescriptions for serotonergic anxiolytics, including selective serotonin reuptake inhibitors, serotonin and norepinephrine reuptake inhibitors, and buspirone.

For further details on variable construction see Appendix 2 of the supplement.

### Statistical Analysis

We constructed separate time-varying Cox proportional hazards regression models for each cohort to examine the relationships between incident co-prescription and the independent variables, both time invariant and time varying. Subsequent 30-day prescribing periods (patient-months) were the units of analysis. Overall model fit was assessed using the Akaike information criterion.

Unlike PDMP models, OLDW models also included the OLDW-exclusive independent variables listed above but otherwise followed the same modeling process as that for the PDMP cohort. To account for the effects of non-benzodiazepine anxiety pharmacotherapy, OLDW models included an interaction term for patients with a diagnosis of depression or anxiety at baseline who also filled prescriptions for serotonergic anxiolytics during follow-up.

Data preparation and analyses were conducted using SAS 9.4.

## RESULTS

The PDMP cohort included 617,946 patients accounting for 13,309,647 patient-months. The OLDW cohort comprised 223,885 patients for 5,277,909 total patient-months. Median (IQR) months of follow-up in the PDMP and OLDW cohorts were 26^17^ and 18,^29^ respectively.

### Descriptive Results

Of the 617,946 patients in the PDMP cohort, 61,168 (9.9%) developed incident co-prescription during the follow-up period with an incidence rate of 4.6 per 1000 patient-months. In the OLDW cohort of 223,885 patients, 20,425 (9.1%) developed co-prescription during the follow-up period with an incidence rate of 3.9 per 1000 patient-months.

Patients with co-prescription were more likely to be female and have higher average MME during baseline (Table 1). Patients with co-prescription were also more likely to be prescribed long-acting opioids, stimulants, have overlapping opioid prescriptions, and have overlapping opioid and non-benzodiazepine sedative prescriptions in the previous 180-day period. Patients with co-prescription were also more likely to experience dose increases over prior short-term (60-day) and long-term (180-day) periods but were less likely to have stable opioid dose trajectories. Patients in the OLDW cohort with co-prescription were more likely to have prior diagnoses for anxiety, depression, substance use disorder, and psychosis; they were also more likely to receive a prescription for serotonergic anxiolytics in the previous 180-day period. Descriptive results for Elixhauser comorbidities are presented in the supplement (Appendix 3).

### Adjusted Analyses

Adjusted analysis of the PDMP cohort revealed that male sex (adjusted hazard ratio [aHR]: 0.70, 95% CI: 0.69–0.71) and older age (relative to age 18–39 years) were associated with reduced risk for co-prescription (Table 2). A higher average baseline daily opioid dose, especially in the highest dose group of > 150 MME (aHR: 1.74, 95% CI: 1.67–1.81), and having 4 or more opioid prescribers (aHR: 1.09, 95% CI: 1.06–1.12) during the baseline period were associated with increased risk for co-prescription.

Long-term (180-day) decreases in 30-day opioid dose (aHR: 0.81, 95% CI: 0.78–0.85) and discontinuation of opioids, particularly sustained long-term discontinuation (aHR: 0.28, 95% CI: 0.25–0.30), were associated with decreased risk for co-prescription. Alternatively, overlapping opioid fills (aHR: 1.33, 95% CI: 1.30–1.36), highly variable opioid dosing (aHR: 1.15, 95% CI: 1.12–1.17), and both short-term (60-day) (aHR: 1.20, 95% CI: 1.17–1.23) and long-term (aHR: 1.13, 95% CI: 1.11–1.16) increases in opioid dose were associated with increased risk for co-prescription. Receipt of any buprenorphine prescription during follow-up (as compared to non-initiation) was associated with increased risk for co-prescription, though the strength of association differed slightly depending on whether patients had (aHR: 1.68, 95% CI: 1.49–1.89) or did not have (aHR: 1.35, 95% CI: 1.18–1.56) an active buprenorphine prescription during the previous 30 days. Overlapping fills of opioids with (a) non-benzodiazepine sedative prescriptions (aHR: 1.69, 95% CI: 1.65–1.72) and (b) prescription psychostimulants (aHR: 1.45, 95% CI: 1.40–1.50) were also associated with increased risk for co-prescription.

Adjusted analysis of the OLDW cohort (Table 3) revealed mostly similar associations but with some differences; patients with 4 or more prescribers (aHR: 0.86, 95% CI: 0.81–0.92) and patients with long-acting opioids in the previous 180 days (aHR: 0.90, 95% CI: 0.86–0.95) had reduced risk for co-prescription (versus increased risk in the PDMP cohort). Unlike in the PDMP cohort, long-term high variability in dose (aHR: 0.98, 95% CI: 0.94–1.03) and increasing long-term dose trajectory (aHR: 0.99, 95% CI: 0.95–1.03) were not associated with co-prescription.

The OLDW cohort model also assessed associations between patients’ clinical characteristics and co-prescription. A history of drug overdose at baseline was associated with increased risk for co-prescription (aHR: 1.25, 95% CI: 1.10–1.42). Additionally, baseline anxiety (aHR: 2.16, 95% CI: 2.06–2.27) and depression (aHR: 1.16, 95% CI: 1.09–1.23) diagnoses without prior fills for serotonergic anxiolytics were risk factors for co-prescription. However, patients with anxiety (aHR: 0.63, 95% CI: 0.59–0.67) or depression (aHR: 0.85, 95% CI: 0.79–0.92) had reduced risk for co-prescription if they also had prior fills for serotonergic anxiolytics.

Adjusted hazard ratios for independent variables not presented in Tables 2 and 3 of the manuscript can be found in the supplement (PDMP: Appendix 4; OLDW: Appendix 5).

### Sensitivity Analysis

Sensitivity analyses with the longer-term definition for co-prescription, a 60-day overlap of opioids and benzodiazepines in a 90-day period, revealed nearly identical findings to our primary analyses. Incidence rates were approximately 1.9 per 1000 person-months in both cohorts; descriptive findings are presented in Appendix 3 and 6. Differences between model estimates for the two outcomes were unremarkable in both cohorts (presented in Appendix 4 and 5).

## DISCUSSION

This study examined two separate cohorts of patients on prescribed long-term opioids—a statewide California cohort (PDMP) and a national commercially insured or Medicare Advantage cohort (OLDW)—to identify factors associated with incident benzodiazepine and opioid co-prescription. Findings were largely consistent across these two different and largely complementary cohorts. Among patients prescribed long-term opioids without benzodiazepines, the cumulative incidence of co-prescription over a mean follow-up of nearly 2 years was considerable and similar across both cohorts; 9.9% in the PDMP cohort and 9.1% in the OLDW cohort. Despite evidence of reduced co-prescription following the published 2016 Centers for Disease Control and Prevention Guidelines^9^ and the boxed warning issued by the US Food and Drug Administration in the same year,^20^ our findings indicate that incident co-prescription remains common among US patients prescribed long-term opioids.

Notably, patients with higher opioid doses during baseline were at subsequent elevated risk for co-prescription. Patients on a higher dose likely have greater difficulty managing their chronic pain which, in turn, may result in pain-associated psychological distress,^21^ and therefore precipitate subsequent benzodiazepine co-prescription.^22,23^ Additionally, benzodiazepines may be prescribed to control muscle spasms as treatment for chronic pain attributed to muscle spasm, particularly among surgical patients.^24,25^

Patients with prior short-term (60-day) changes in daily opioid dose were at significantly increased risk for co-prescription in both cohorts. Short-term reductions may reflect opioid tapers, which are associated with subsequent mental health crises and overdose.^10,26^ Possibly due to subsequent withdrawal-related dysphoria and anxiety which then lead to benzodiazepine co-prescription. In contrast, prior long-term (180-day) decreases in dose were significantly associated with decreased risk for co-prescription in both cohorts. Overall, these findings suggest that opioid dose reduction may pose a short-term hazard for co-prescription, but that longer-term risk is reduced if patients can be successfully transitioned to lower opioid doses.

In both cohorts, patients who received buprenorphine indicated to treat opioid use disorder were significantly more likely to develop incident co-prescription. Findings were comparable across cohorts even with analysis of the OLDW cohort controlling for substance use disorder and anxiety diagnoses. Among patients who received buprenorphine after cohort entry, those with active buprenorphine prescriptions in the prior 30 days were more likely to be co-prescribed than those without. These findings suggest that patients who initiate and remain on buprenorphine treatment are more likely to be co-prescribed, possibly due to prescribers viewing patients actively prescribed buprenorphine as at reduced risk for overdose.^27^

Analysis in the OLDW cohort revealed anxiety diagnosis during baseline as a risk factor for co-prescription, but that when these patients received serotonergic anxiolytics to treat anxiety their risk for co-prescription was reduced. We found a similar but attenuated effect for depression diagnoses. These findings suggest that treatment of anxiety and depression disorders with serotonergic anxiolytics may reduce the likelihood of co-prescription for patients prescribed long-term opioids.

Although our study was not designed to test causation, the consistent findings across two complementary studies do suggest implications for clinical care. To prevent transition to co-prescription among patients with long-term opioid therapy, strategies to reduce pain-associated anxiety and distress are critical. Beyond use of non-benzodiazepine medications, the Anxiety and Depression Association of America recommends cognitive-behavioral therapy, relaxation techniques, and alternative treatments (e.g., acupuncture). Additionally, lifestyle changes, specifically avoiding alcohol and caffeine, will improve sleep quality and reduce need for insomnia-related benzodiazepine use.^28^ For patients undergoing opioid tapers, following best practices can reduce subsequent anxiety; such as initiating tapers in a slow, deliberative, and patient-centered manner, coordinating tapers with adjuvant treatments to mitigate withdrawal symptoms, and multidisciplinary coordinated care to ensure psychosocial support to reduce spikes in patient anxiety and pain.^29^

This study has limitations. First, although the OLDW cohort included outpatient diagnoses, neither cohort includes information regarding clinical rationale for prescribing patterns or patient access to care. Second, our baseline period may not capture patients with benzodiazepine prescribing gaps greater than 6 months. However, we believe a 6-month lookback period is sufficient to exclude most patients who use benzodiazepines semi-regularly (2 or more prescriptions per year). Third, these data do not capture illicit substance use, which may be associated with co-prescription. Finally, analysis of the OLDW cohort is limited by the exclusion of patients who were not continuously enrolled throughout baseline. Concerns are tempered, however, due to the consistent findings for patient factors that were also analyzed with the PDMP cohort and not subject to enrollment limitations.

In statewide and national cohorts, we found that incident benzodiazepine co-prescription remains common among patients prescribed long-term opioids. We also identified key risk factors for co-prescription, including female sex, high opioid dose, short-term changes in opioid dose, buprenorphine initiation, and anxiety or depression not treated with serotonergic anxiolytics. Our findings indicate the need for multidisciplinary care for patients treated with long-term opioids to avoid transition to co-prescription with benzodiazepines.

## Supplementary Material

Supplementary Appendix

**Supplementary Information** The online version contains supplementary material available at https://doi.org/10.1007/s11606-025-09712-2.

## Figures and Tables

**Table 1:** 30-Day Observation Period Characteristics Within Each Cohort, by Outcome Status (Co-prescription)

	PDMP (N = 13,309,647)			OLDW (N = 5,277,909)		
		
		Co-prescription status			Co-prescription status	
			
	Total	Yes (*n* = 61,168)	No (*n* = 13,248,479)	Total	Yes (*n* = 20,425)	No (*n* = 5,257,484)

Independent variables	%	%	%	%	%	%
Patient age at study entry						
18–39	10.4	11.4	10.4	4.9	6.1	4.9
40–65	58.4	57.9	58.4	50.1	55.2	50.1
> 65	31.2	30.8	31.2	45.0	38.7	45.0
Patient sex						
Female	52.4	61.7	52.3	58.0	65.7	58.0
Male	47.6	38.3	47.7	42.0	34.3	42.0
Insurance status						
Commercial insurance				29.2	31.1	29.2
Any Medicare Advantage				70.8	68.9	70.8
Rural–urban commuting area designation*						
Metropolitan				80.3	80.0	80.3
Micropolitan				10.8	11.5	10.8
Small town or rural area				8.9	8.4	8.9
Unknown				0.1	0.1	0.1
Census block median educational level*						
Less than or high school diploma				37.8	38.0	37.8
Less than bachelor’s degree				52.6	52.1	52.6
Bachelor’s degree plus				7.9	8.3	7.9
Unknown				1.7	1.7	1.7
Census block median household income*						
< $40,000				40.8	40.1	40.8
$40,000–$74,999				27.8	27.0	27.8
$75,000–$124,999				19.0	18.9	19.0
$125,000–$199,999				5.5	6.0	5.5
≥ $200,000				2.2	2.6	2.2
Unknown				4.7	5.4	4.7
Baseline^†^ average daily dose (MME)						
1–19	31.4	22.5	31.4	40.5	26.6	40.5
20–49	38.8	39.3	38.8	35.0	39.2	35.0
50–89	14.4	17.3	14.3	11.9	16.5	11.9
90–149	7.1	9.3	7.0	5.8	8.3	5.8
150 or more	8.4	11.7	8.4	6.7	9.4	6.7
Baseline^†^ total opioid prescribers						
1 prescriber	45.8	43.3	45.9	57.3	56.7	57.3
2 prescribers	31.0	30.2	31.0	27.8	27.3	27.8
3 prescribers	14.1	14.9	14.1	9.8	10.3	9.8
4 or more prescribers	9.0	11.5	9.0	4.6	5	4.5
Predominant opioid type during baseline^†^						
Codeine	3.0	2.6	3.0	2.5	2.4	2.5
Fentanyl	3.0	4.5	3.0	3.0	3.9	3.0
Hydrocodone	52.1	49.1	52.1	35.9	36.3	35.9
Hydromorphone	1.1	1.6	1.1	0.9	1.4	0.9
Methadone	3.2	3.5	3.2	1.8	1.8	1.8
Morphine	6.0	6.3	6.0	4.3	4.9	4.3
Oxycodone	16.2	20.6	16.2	24.1	30.6	24.1
Tramadol	13.9	10.2	13.9	25.0	16.1	25.0
Other (e.g., multiple opioid types)	1.5	1.7	1.5	2.5	2.8	2.5
Any non-fatal overdose during baseline^‡^				0.6	1.2	0.6
Mental health diagnoses^†^^§^						
Anxiety				15.3	30.3	15.2
Alcohol use disorder				1.3	1.5	1.3
Depression				16.3	24.2	16.3
Substance use disorder				7.7	10.0	7.7
Psychoses				4.6	8.1	4.6
Buprenorphine status						
No buprenorphine	99.2	99.2	99.2	99.2	99.3	99.2
Buprenorphine initiation and no use prior 30 days	0.4	0.3	0.4	0.4	0.3	0.4
Buprenorphine initiation and use prior 30 days	0.4	0.5	0.4	0.4	0.5	0.4
Any long-acting opioid fill^¶^	20.2	28.3	20.2	15.9	22.2	15.9
Any multiple opioid fill overlap^¶^	26.5	40.2	26.5	20.6	30.2	20.5
Any opioid/non-benzodiazepine sedative overlap^¶^	13.4	24.1	13.3	37.3	46.2	37.3
Any psychostimulant fill^¶^	3.4	5.9	3.4	2.9	5.5	2.9
Any serotonergic anxiolytic^∥^ fill^¶^				34.3	47.4	34.2
Short-term (60-day) dose (MME) trajectory						
Decrease	9.1	11.7	9.1	7.5	9.1	7.5
Stable	81.5	73.9	81.5	84.9	80.4	85.0
Increase	9.4	14.4	9.4	7.6	10.5	7.5
Long-term (180-day) dose trajectory						
Decrease	9.5	7.2	9.5	7.8	5.8	7.8
Stable	71.0	65.6	71.0	73.3	71.0	73.3
Increase	19.6	27.2	19.5	19.0	23.2	19.0
Long-term (180-day) dose variability						
Low	41.3	31.8	41.3	48.1	41.2	48.1
Moderate	34.1	38.2	34.1	30.8	35.5	30.8
High	24.6	30.1	24.6	21.1	23.3	21.1
Opioid discontinuation status^#^						
No discontinuation	83.6	94.5	83.5	77.6	93.1	77.5
Low-dose short-term discontinuation	5.4	2.1	5.5	8.6	3.4	8.6
High-dose short-term discontinuation	0.5	0.3	0.5	0.4	0.3	0.4
Any dose long-term discontinuation	7.4	1.0	7.4	9.3	1.3	9.3
Resumption from prior discontinuation	3.1	2.1	3.1	4.2	1.9	4.2

Empty cells indicate variables not available for analysis in that cohort

*PDMP*, California prescription drug monitoring program data; *OLDW*, Optum Labs Data Warehouse; *MME*, milligram morphine equivalents

* Variables measured at census block level

† Baseline: 180-day period prior to study entry

‡ Baseline: 365-day period prior to study entry

§ Identified from AHRQ Elixhauser comorbidity index

∥ Selective serotonin reuptake inhibitors, serotonin and norepinephrine reuptake inhibitors, and buspirone

¶ Baseline and/or follow-up: 180-day period prior to patient-month

# Low dose: < 50 MME daily, high dose: ≥ 50 MME daily, short term: in 60 days, long term: in 180 days

**Table 2:** Time-Varying Cox Proportional Model Derived Adjusted Hazard Ratios for Co-prescription, California Prescription Drug Monitoring Program (PDMP) Cohort, 2016–2018

Independent variables	aHR	95% CI	*p*-value

Patient age at study entry (Ref = 18–39)			
40–65	**0.89**	**0.87–0.92**	**< 0.001**
Older than 65	**0.93**	**0.90–0.96**	**< 0.001**
Patient sex (Ref = female)			
Male	**0.70**	**0.69–0.71**	**< 0.001**
Baseline* average daily dose (MME) (Ref = 1–19)			
20–49	**1.35**	**1.32–1.38**	**< 0.001**
50–89	**1.53**	**1.48–1.58**	**< 0.001**
90–149	**1.65**	**1.59–1.72**	**< 0.001**
150 or more	**1.74**	**1.67–1.81**	**< 0.001**
Baseline* total opioid prescribers (Ref = 1 prescriber)			
2 prescribers	**0.98**	**0.96–1.00**	**0.013**
3 prescribers	1.00	0.98–1.03	0.844
4 or more prescribers	**1.09**	**1.06–1.12**	**< 0.001**
Buprenorphine status (Ref = no buprenorphine)			
Buprenorphine initiation and no use prior 30 days	**1.35**	**1.18–1.56**	**< 0.001**
Buprenorphine initiation and use prior 30 days	**1.68**	**1.49–1.89**	**< 0.001**
Any long-acting opioid fill^†^ (Ref = no)	**1.03**	**1.00–1.06**	**0.046**
Any multiple opioid fill overlap^†^ (Ref = no)	**1.33**	**1.30–1.36**	**< 0.001**
Any opioid/non-benzodiazepine sedative overlap^†^ (Ref = no)	**1.69**	**1.65–1.72**	**< 0.001**
Any psychostimulant fill^†^ (Ref = no)	**1.45**	**1.40–1.50**	**< 0.001**
Short-term (60-day) dose trajectory (Ref = stable)			
Decrease	**1.07**	**1.04–1.10**	**< 0.001**
Increase	**1.20**	**1.17–1.23**	**< 0.001**
Long-term (180-day) dose trajectory (Ref = stable)			
Decrease	**0.81**	**0.78–0.85**	**< 0.001**
Increase	**1.13**	**1.11–1.16**	**< 0.001**
Long-term (180-day) dose variability (Ref = low)			
Moderate	**1.04**	**1.02–1.06**	**0.006**
High	**1.15**	**1.12–1.17**	**< 0.001**
Opioid discontinuation status^‡^ (Ref = no discontinuation)			
Low dose short-term discontinuation	**0.57**	**0.54–0.61**	**< 0.001**
High-dose short-term discontinuation	**0.58**	**0.50–0.67**	**< 0.001**
Any dose long-term discontinuation	**0.28**	**0.25–0.30**	**< 0.001**
Resumption from prior discontinuation	1.05	0.99–1.11	0.083

Model adjusted for year of study entry and predominant opioid type during baseline; estimates not shown but presented in Appendix 4

*aHR*, adjusted hazard ratio; *95% CI*, 95% confidence intervals; *MME*, milligram morphine equivalents

* Baseline: 180-day period prior to study entry

† Baseline and/or follow-up: 180-day period prior to patient-month

‡ Low dose: < 50 MME daily, high dose: ≥ 50 MME daily, short term: in 60 days, long term: in 180 days

**Table 3:** Time-Varying Cox Proportional Model Derived Adjusted Hazard Ratios for Co-prescription, US Sample of Commercially and/or Medicare Advantage Insured (OLDW) Cohort, 2016–2021

Independent variables	aHR	95% CI	*p*-value

Patient age at study entry (Ref = 18–39)			
40–65	**0.94**	**0.89–1.00**	**0.049**
Older than 65	**0.91**	**0.85–0.98**	**0.008**
Patient sex (Ref = female)			
Male	**0.80**	**0.78–0.83**	**< 0.001**
Insurance status (Ref = commercial insurance)			
Any Medicare Advantage	0.97	0.93–1.00	0.082
Baseline* average daily dose (MME) (Ref = 1–19)			
20–49	**1.63**	**1.57–1.69**	**< 0.001**
50–89	**2.08**	**1.97–2.19**	**< 0.001**
90–149	**2.36**	**2.20–2.53**	**< 0.001**
150 or more	**2.66**	**2.47–2.86**	**< 0.001**
Baseline* total opioid prescribers (Ref = single prescriber)			
2 prescribers	**0.90**	**0.87–0.93**	**< 0.001**
3 prescribers	**0.88**	**0.84–0.92**	**< 0.001**
4 or more prescribers	**0.86**	**0.81–0.92**	**< 0.001**
Any non-fatal overdose during baseline^†^ (Ref = none)	**1.25**	**1.10–1.42**	**< 0.001**
Mental health status^‡^ (Ref = no diagnosis or serotonergic anxiolytic^§^ fills^∥^)			
Anxiety diagnosis without serotonergic anxiolytic	**2.16**	**2.06–2.27**	**< 0.001**
Anxiety diagnosis with serotonergic anxiolytic	**0.63**	**0.59–0.67**	**< 0.001**
Depression diagnosis without serotonergic anxiolytic	**1.16**	**1.09–1.23**	**< 0.001**
Depression diagnosis with serotonergic anxiolytic	**0.85**	**0.79–0.92**	**< 0.001**
No diagnosis with serotonergic anxiolytic	**1.51**	**1.46–1.57**	**< 0.001**
Alcohol use disorder diagnosis	**0.89**	**0.79–1.00**	**< 0.001**
Substance use disorder diagnosis	1.01	0.96–1.06	0.719
Psychoses diagnosis	**1.19**	**1.13–1.25**	**< 0.001**
Buprenorphine status (Ref = no buprenorphine)			
Buprenorphine initiation and no use prior 30 days	**1.64**	**1.27–2.11**	**< 0.001**
Buprenorphine initiation and use prior 30 days	**2.10**	**1.71–2.59**	**< 0.001**
Any long-acting opioid fill^∥^ (Ref = no)	**0.90**	**0.86–0.95**	**< 0.001**
Any multiple opioid fill overlap^∥^ (Ref = no)	**1.17**	**1.13–1.21**	**< 0.001**
Any opioid/non-benzodiazepine sedative overlap^∥^ (Ref = no)	**1.05**	**1.02–1.08**	**< 0.001**
Any psychostimulant fill^∥^ (Ref = no)	**1.34**	**1.26–1.42**	**< 0.001**
Short-term (60-day) dose (MME) trajectory (Ref = stable)			
Decrease	**1.06**	**1.01–1.12**	**0.022**
Increase	**1.13**	**1.07–1.18**	**< 0.001**
Long-term (180-day) dose trajectory (Ref = stable)			
Decrease	**0.78**	**0.73–0.84**	**< 0.001**
Increase	0.99	0.95–1.03	0.620
Long-term (180-day) dose variability (Ref = low)			
Moderate	0.97	0.94–1.01	0.142
High	0.98	0.94–1.03	0.480
Opioid discontinuation status^¶^ (Ref = no discontinuation)			
Low-dose short-term discontinuation	**0.76**	**0.70–0.83**	**< 0.001**
High-dose short-term discontinuation	**0.72**	**0.55–0.94**	**0.018**
Any dose long-term discontinuation	**0.30**	**0.26–0.34**	**< 0.001**
Resumption from prior discontinuation	1.01	0.91–1.12	0.884

Model adjusted for year of study entry, area-level characteristics (rurality, median income, median education), and Elixhauser comorbidity; estimates not shown but presented in Appendix 5

*OLDW*, Optum Labs Data Warehouse; *aHR*, adjusted hazard ratio; *95% CI*, 95% confidence intervals; *MME*, milligram morphine equivalents

* Baseline: 180-day period prior to study entry

† Baseline: 365-day period prior to study entry

‡ Identified from AHRQ Elixhauser comorbidity index in the 180-day period prior to study entry

§ Selective serotonin reuptake inhibitors, serotonin and norepinephrine reuptake inhibitors, and buspirone

∥ Baseline and/or follow-up: 180-day period prior to patient-month

¶ Low dose: < 50 MME daily, high dose: ≥ 50 MME daily, short term: in 60 days, long term: in 180 days

## Data Availability

Data utilized for this study were obtained through data use agreements between the authors and the curators of these data sources, and are therefore not available to third parties.
