# Supplementary material for: Predictors of Incident Benzodiazepine Co-prescription Among Patients Prescribed Long-term Opioids
Source: J Gen Intern Med. 2025 Jul 16;41(4):1030–40. doi: 10.1007/s11606-025-09712-2 (PMC12370231; doi:10.1007/s11606-025-09712-2)
Supplement: Supplementary file 1 — Supplementary file1 (DOCX 95 KB) [file 11606_2025_9712_MOESM1_ESM.docx]

**Supplemental material for *Predictors of incident benzodiazepine co-prescription among patients prescribed long term opioids***

**Table of Contents**

| **Section** | | **Page** |
| --- | --- | --- |
| Appendix 1 | Trajectory variable construction and definitions | 2 |
| Appendix 2 | Independent variable construction details | 4 |
| Appendix 3 | Elixhauser comorbidity distributions in OLDW cohort by long-term co-prescription definition (20-day overlap in 30-day period versus 60-day overlap in 90-day period) | 6 |
| Appendix 4 | Comparison of time-varying Cox proportional models for primary (20-day overlap in 30-day period) and secondary (60-day overlap in 90-day period) definitions of long-term co-prescription, California PDMP cohort, 2016—2018 | 7 |
| Appendix 5 | Comparison of Time-varying Cox Proportional Models for primary (20-day overlap in 30-day period) and secondary (60-day overlap in 90-day period) definitions of long-term co-prescription, US sample of commercially and/or Medicare Advantage insured (OLDW) cohort, 2016—2021 | 9 |
| Appendix 6 | Observation period (30-day) characteristics within each cohort, by secondary definition (60-day overlap in 90-day period) for long-term co-prescription | 12 |
| References | Supplement References | 15 |

**Appendix 1: Trajectory variable construction and definitions**

*Short-term MME trajectory*

The short-term milligram morphine equivalent (MME) trajectory for the observation period (Month 0) is based on the change in average daily MME between the prior two months (Months -2 [M-2] and -1 [M-1]). “Months” correspond to 30-day windows with Month 0 equivalent to the period between Days 0 to -29, Month -1 equivalent to Days -30 to -59, and Month -2 equivalent to Days -60 to -89. Calculations were done as follows:

- Absolute Change:

$${MME}_{M-1}-{MME}_{M-2}$$

- Relative Change:

$$\frac{{MME}_{M-1}-{MME}_{M-2}}{{MME}_{M-1}}*100$$

Definitions depended on the daily MME average in Month -1. Patients were defined as stable, increasing, or decreasing with details in the table below:

| **MME in M-1** | **Threshold for decreasing short-term trajectory**  (absolute or relative change in MME between Months -1 and -2) | **Threshold for increasing short-term trajectory**  (absolute or relative change in MME between Months -1 and -2) |
| --- | --- | --- |
| 0 | None | 10 |
| 1-19 | Discontinued (to 0 MME) | 10 |
| 20-49 | 10 | 10 |
| 50-89 | 10 | 10 |
| 90-149 | 15% | 15% |
| ≥150 | 15% | 15% |

*Long-term MME trajectory*

To identify long-term trajectory of opioid use in the observation period (Month 0), we first performed a descriptive analysis of mean slope in opioid doses (representing the average monthly change in dose in MME) across the initial six 30-day periods (180 days, Months -1 through -6) of follow-up. For comparison, we also examined the mean slopes in MME during the 180-day baseline period prior to cohort entry. To account for high variability in dosing, we used Poisson regression models, generalized linear models with a Poisson distribution and log link, to estimate unadjusted patient-specific slopes measuring change per month in the log of the expected value of the dose. We then computed percent change per month in expected MME from estimated slopes using the following function:

$$=\left( e^{\beta}-1 \right)*100$$

Then stratified the sample by baseline MME categories (during the baseline 180-days of long-term opioid therapy) in order to compute means, medians, and percentiles. Based on the approximately the 10-20^th^ and 80-90^th^ percentiles, we identified thresholds for dose changes that we believed were clinically meaningful. For patients with no prescribed opioids during the 180-day period, we classified the dose as stable.

Patients were defined as stable, increasing, or decreasing with details in the table below:

| **Mean prior 180-day MME** | **Threshold for decreasing long-term trajectory**  (monthly average % change between Months -1 and -6) | **Threshold for increasing long-term trajectory**  (monthly average % change between Months -1 and -6) |
| --- | --- | --- |
| 0 | Stable* | Stable* |
| 1-19 | 25% | 5% |
| 20-49 | 15% | 4% |
| 50-89 | 10% | 3% |
| 90-149 | 10% | 2% |
| ≥150 | 10% | 2% |

* Patients prescribed no opioids during the prior 180-day period (0 MME) were defined as stable.

*Long-term MME variability*

MME variability was determined by calculating the standard deviation for average daily MME for the 6 months (Months -1 to -6) prior to the observation period (Month 0). Categorization was based on the average MME over the entire 6-month period and classified as low, moderate, or high. Definitions varied by cohort. Details appear in the table below:

| **Variability Definition** | **Mean prior 180-day MME** | **Prescription Drug Monitoring Program** | **Optum Labs Data Warehouse** |
| --- | --- | --- | --- |
| Low | 1-19 | < 2.0 | < 2.0 |
|  | 20-49 | < 3.5 | < 3.5 |
|  | 50-89 | < 6.3 | < 6.3 |
|  | 90-149 | < 10 | < 10 |
|  | ≥150 | < 21 | < 12 |
| Moderate | 1-19 | 2 ≤ SD ≤ 5 | 2 ≤ SD ≤ 5 |
|  | 20-49 | 3.5 ≤ SD ≤ 10 | 3.5 ≤ SD ≤ 10 |
|  | 50-89 | 6.3 ≤ SD ≤ 20 | 6.3 ≤ SD ≤ 20 |
|  | 90-149 | 10 ≤ SD ≤ 30 | 10 ≤ SD ≤ 30 |
|  | ≥150 | 21 ≤ SD ≤ 60 | 12 ≤ SD ≤ 45 |
| High | 1-19 | >5.0 | >5.0 |
|  | 20-49 | >10 | >10 |
|  | 50-89 | >20 | >20 |
|  | 90-149 | >30 | >30 |
|  | ≥150 | >60 | >45 |

**Appendix 2: Independent variable construction details**

We derived time-invariant and time-varying covariates in our 30-day time-varying models. The time-invariant refers to variables with values fixed for all 30-day intervals while time-varying refers to those variables with dynamic values during the follow-up period.

***Time-invariant independent variables:***

*Sociodemographics*

Patient-level variables included age and sex while area-level variables included rurality of residence, insurance status, median educational level, and median income range. Age was divided to 18-39, 40-65, and more than 65 years. Age and sex excluded, sociodemographic covariates were derived from member coverage data and consumer profiles in the Optum Labs Data Warehouse. Patient insurance status was categorized as those with exclusively commercial insurance and those with any Medicare Advantage (combinations of the two were classified as Medicare Advantage). Rurality of residence was derived from the Rural-Urban Commuting Area codes using three categories: metropolitan, micropolitan, and small town or rural area. Median educational level (less than or high school diploma, less than bachelor’s degree, and bachelor’s degree plus) and median income range (i.e., <$40,000, $40,000-$74,999, $75,000-$124,999, $125,000-$199,999, and ≥$200,000) were derived from patient census block of residence.

*Average monthly MME dose*

The MME daily dose is calculated using a standard conversion factor to multiply total prescribed dose and divide by the total supply days of prescription. We first calculated average daily MME dose for each month of baseline, and then calculated an average of daily dose across the last 6 months of each patient’s baseline period; with patient categorized as 1-19, 20-49, 50-89, 90-149, and 150 or more MME.

*Number of opioid prescribers*

We identified the number of unique opioid prescribers during each patient’s baseline period, and categorized them as having 1, 2, 3, and 4 or more opioid prescribers.

*Predominant opioid type*

We determined the predominant opioid type using the maximum total MME during the 180-day baseline period. Opioid type was categorized as “Other” if there were more than one drug type with the same maximum total MME, or if the predominant drug type was among those that made up less than 1% of prescriptions in the data, specifically: Butorphanol, Dihydrocodeine, Levorphanol, Meperidine, Opium, Oxymorphone, Pentazocine, and Tapentadol. Categories of predominant opioid type were Hydrocodone, Codeine, Fentanyl, Hydromorphone, Methadone, Morphine, Oxycodone, Tramadol, and Other.

*Non-fatal overdose events* (OptumLabs Data Warehouse Only)

Overdose events were defined as emergency department visits or inpatient hospital admissions for non-fatal drug overdoses occurring during the 12-month baseline period. Overdose events were identified using International Classification of Diseases, Clinical Modification, Tenth Revision codes for this outcome by augmenting the definition for “all-drug overdose” specified in CDC drug overdose surveillance guidelines:(1) including any prescription and/or illicit opioid (ICD-10-CM codes: T400-T404, T4060, T4069), benzodiazepine (ICD-10-CM codes: T424), and illicit psychostimulant (ICD-10-CM codes: T405, T4360, T4362, T4364, T4369) overdoses.

*Comorbidities* (OptumLabs Data Warehouse Only)

Based on methods utilized in prior work,(2) the Elixhauser comorbidity index was used to identify specific patient’s comorbidities by analyzing medical claims data during the 180 days prior to the index date. Patient anxiety was derived from medical claims via ICD-10-CM codes: F064, F400-F410, F449, F458, F488-F489, R452, R455-R457, F41-F43, and F99. A list of all comorbidities and their corresponding effect estimates are shown in Appendix 1.

***Time-varying independent variables:***

*Buprenorphine status*

Patients with fills for buprenorphine, indicated for opioid use disorder, during baseline were excluded from the study, however we constructed a variable to identify buprenorphine initiation during the follow-up period. Additional to initiation, patients were also categorized based on if they had any active prescriptions for buprenorphine in the preceding month. Categories included, no buprenorphine initiation, buprenorphine initiated but no use in prior 30 days, buprenorphine initiation and use in prior 30 days.

*Prescriptions drugs overlap days*

We assessed several patterns for prescription overlaps: (1) multiple opioid types, (2) opioid and other sedatives (including, Z-drugs (zolpidem, eszopiclone, and zaleplon) and carisoprodol, and, for OLDW data only, gabapentenoids), (3) stimulants, and (4) non-benzodiazepine anxiolytics (i.e., selective serotonin reuptake inhibitors (SSRIs), serotonin and norepinephrine reuptake inhibitors (SNRIs), and buspirone) during the prior 6 months. Non-controlled substances, including gabapentenoids, SSRI, SNRI, and buspirone, are not identifiable in the PDMP cohort.

*Discontinuation Status*

To identify patient discontinuation status, we began assessment of discontinuation in the first month of their follow-up period. The status included, never discontinued, short-term discontinuation from low dose (1-49 MME), short-term discontinuation from high MME dose (≥50 MME), long-term discontinuation, and any resumption from a discontinuation. Short-term discontinuation was defined as 0 MME for two to five consecutive 30-day periods, and long-term discontinuation was defined as 0 MME for at least six consecutive 30-day periods. Resumption was achieved if there were two consecutive 30-day periods with mean daily MME > 10 after any short-term or long-term discontinuation.

**Appendix 3: Elixhauser comorbidity distributions in OLDW cohort by long-term co-prescription definition (20-day overlap in 30-day period versus 60-day overlap in 90-day period)**

|  | **20/30 Days with Overlap** | | |  | **60-90 Days with Overlap** | | |
| --- | --- | --- | --- | --- | --- | --- | --- |
|  |  | **Co-prescription Status** | |  |  | **Co-prescription Status** | |
|  | **Total** | **Yes**  (n=) | **No**  (n=) |  | **Total** | **Yes**  (n=) | **No**  (n=) |
| **Elixhauser comorbidity indicators** | **%** | **%** | **%** |  | **%** | **%** | **%** |
| Acquired immune deficiency syndrome | 0.4 | 0.4 | 0.4 |  | 0.4 | 0.5 | 0.4 |
| Deficiency anemias | 11.8 | 12.8 | 11.8 |  | 11.8 | 12.8 | 11.8 |
| Autoimmune conditions | 8.2 | 9.3 | 8.2 |  | 8.3 | 8.7 | 8.3 |
| Chronic blood loss anemia | 0.9 | 0.9 | 0.9 |  | 0.9 | 0.9 | 0.9 |
| Cerebrovascular disease | 5.1 | 5.5 | 5.1 |  | 5.1 | 5.5 | 5.1 |
| Coagulopathy | 1.6 | 1.7 | 1.6 |  | 1.6 | 1.7 | 1.6 |
| Dementia | 3.3 | 3.1 | 3.3 |  | 3.2 | 2.3 | 3.2 |
| Diabetes with chronic complications | 17.8 | 16.6 | 17.8 |  | 17.7 | 16.4 | 17.7 |
| Diabetes without chronic complications | 11.4 | 10.8 | 11.4 |  | 11.4 | 10.8 | 11.4 |
| Congestive heart failure | 6.9 | 7.3 | 6.9 |  | 6.8 | 6.9 | 6.8 |
| Hypertension, complicated | 9.3 | 8.8 | 9.3 |  | 9.2 | 8.0 | 9.2 |
| Hypertension, uncomplicated | 52.1 | 50.0 | 52.1 |  | 52.0 | 50.5 | 52.0 |
| Liver disease, mild | 4.2 | 5.0 | 4.2 |  | 4.2 | 5.4 | 4.2 |
| Liver disease, moderate to severe | 0.4 | 0.5 | 0.4 |  | 0.4 | 0.4 | 0.4 |
| Chronic pulmonary disease | 19.4 | 23.8 | 19.4 |  | 19.5 | 24.9 | 19.4 |
| Neurological disorders affecting movement | 3.2 | 4.3 | 3.2 |  | 3.2 | 4.0 | 3.2 |
| Other neurological disorders | 2.3 | 3.3 | 2.3 |  | 2.3 | 3.6 | 2.3 |
| Seizures and epilepsy | 1.9 | 3.0 | 1.9 |  | 2.0 | 3.3 | 2.0 |
| Obesity | 18.7 | 19.8 | 18.7 |  | 18.8 | 19.6 | 18.8 |
| Paralysis | 1.6 | 1.8 | 1.6 |  | 1.6 | 1.7 | 1.6 |
| Peripheral vascular disease | 10.3 | 9.9 | 10.3 |  | 10.2 | 9.3 | 10.3 |
| Pulmonary circulation disease | 1.4 | 1.6 | 1.4 |  | 1.4 | 1.5 | 1.4 |
| Renal failure, moderate | 8.0 | 6.8 | 8.0 |  | 8.0 | 6.4 | 8.0 |
| Renal failure, severe | 1.3 | 1.0 | 1.3 |  | 1.3 | 0.9 | 1.3 |
| Hypothyroidism | 14.9 | 17.0 | 14.9 |  | 15.0 | 16.7 | 15.0 |
| Other thyroid disorders | 2.5 | 3.1 | 2.5 |  | 2.6 | 2.7 | 2.6 |
| Peptic ulcer with bleeding | 1.0 | 1.3 | 1.0 |  | 1.0 | 1.2 | 1.0 |
| Valvular disease | 5.0 | 5.5 | 5.0 |  | 5.0 | 5.1 | 5.0 |
| Weight loss | 2.5 | 2.9 | 2.5 |  | 2.5 | 3.0 | 2.5 |
| OLDW: Optum Labs Data Warehouse | | | | | | | |

**Appendix 4: Comparison of time-varying Cox proportional models for primary (20-day overlap in 30-day period) and secondary (60-day overlap in 90-day period) definitions of long-term co-prescription,** **California PDMP cohort, 2016—2018**

|  | **20 out of 30 days with overlap**  **(n=61 168)** | | **60 out of 90 days with overlap**  **(n=26 721)** | |
| --- | --- | --- | --- | --- |
| **Independent variables** | **aHR** | **95% CI** | **aHR** | **95% CI** |
| Patient age at study entry, (Ref = 18-39) |  |  |  |  |
| 40-65 | **0.89** | **0.87-0.92** | **0.80** | **0.77-0.83** |
| More than 65 | **0.93** | **0.90-0.96** | **0.72** | **0.69-0.75** |
| Patient sex, (Ref = Female) |  |  |  |  |
| Male | **0.70** | **0.69-0.71** | **0.74** | **0.72-0.75** |
| Baseline^a^ average daily dose (MME), (Ref = 1-19) |  |  |  |  |
| 20-49 | **1.35** | **1.32-1.38** | **1.40** | **1.35-1.45** |
| 50-89 | **1.53** | **1.48-1.58** | **1.62** | **1.54-1.69** |
| 90-149 | **1.65** | **1.59-1.72** | **1.89** | **1.78-2.01** |
| 150 or more | **1.74** | **1.67-1.81** | **2.08** | **1.95-2.20** |
| Baseline^a^ total opioid prescribers, (Ref = 1 Prescriber) |  |  |  |  |
| 2 prescribers | **0.98** | **0.96-1.00** | 0.97 | 0.95-1.00 |
| 3 prescribers | 1.00 | 0.98-1.03 | 1.02 | 0.98-1.05 |
| 4 or more prescribers | **1.09** | **1.06-1.12** | **1.12** | **1.07-1.16** |
| Predominant opioid type during baseline^a^, (Ref = Hydrocodone) |  |  |  |  |
| Codeine | 0.97 | 0.92-1.02 | 1.04 | 0.96-1.12 |
| Fentanyl | **0.95** | **0.90-0.99** | **0.82** | **0.76-0.88** |
| Hydromorphone | 1.02 | 0.95-1.08 | 0.92 | 0.84-1.02 |
| Methadone | **0.82** | **0.78-0.86** | **0.81** | **0.75-0.87** |
| Morphine | **0.80** | **0.77-0.83** | **0.75** | **0.71-0.80** |
| Oxycodone | 1.01 | 0.99-1.04 | 0.97 | 0.93-1.00 |
| Tramadol | **0.87** | **0.84-0.89** | **0.89** | **0.85-0.93** |
| Other (e.g., multiple opioid types) | **0.81** | **0.76-0.86** | **0.71** | **0.64-0.78** |
| Year of cohort entry (Ref = 2016) |  |  |  |  |
| 2017 | **1.47** | **1.44-1.50** | **1.29** | **1.29-1.36** |
| 2018 | **1.34** | **1.30-1.38** | **0.92** | **0.87-0.98** |
| Buprenorphine status, (Ref = No buprenorphine) |  |  |  |  |
| Buprenorphine initiation and no use prior 30 days | **1.35** | **1.18-1.56** | **1.26** | **1.03-1.55** |
| Buprenorphine initiation and use prior 30 days | **1.68** | **1.49-1.89** | **1.96** | **1.68-2.29** |
| Any long-acting opioid fill^b^, (Ref = No) | **1.03** | **1.00-1.06** | **0.94** | **0.91-0.98** |
| Any multiple opioid fill overlap^b^, (Ref = No) | **1.33** | **1.30-1.36** | **1.42** | **1.38-1.47** |
| Any opioid/non-benzodiazepine sedative overlap^b^, (Ref = No) | **1.69** | **1.65-1.72** | **2.04** | **1.99-2.10** |
| Any psychostimulant fill^b^, (Ref = No) | **1.45** | **1.40-1.50** | **1.41** | **1.34-1.49** |
| Short-term (60-day) dose trajectory, (Ref = Stable) |  |  |  |  |
| Decrease | **1.07** | **1.04-1.10** | **1.07** | **1.03-1.11** |
| Increase | **1.20** | **1.17-1.23** | **1.10** | **1.06-1.14** |
| Long-term (180-day) dose trajectory, (Ref = Stable) |  |  |  |  |
| Decrease | **0.81** | **0.78-0.85** | **0.86** | **0.81-0.91** |
| Increase | **1.13** | **1.11-1.16** | **1.18** | **1.15-1.22** |
| Long-term (180-day) dose variability, (Ref = Low) |  |  |  |  |
| Moderate | **1.04** | **1.02-1.06** | 1.01 | 0.98-1.04 |
| High | **1.15** | **1.12-1.17** | **1.13** | **1.09-1.17** |
| Opioid discontinuation status,^c^ (Ref = No discontinuation) |  |  |  |  |
| Low dose short-term discontinuation | **0.57** | **0.54-0.61** | **0.49** | **0.45-0.54** |
| High dose short-term discontinuation | **0.58** | **0.50-0.67** | **0.67** | **0.56-0.81** |
| Any dose long-term discontinuation | **0.28** | **0.25-0.30** | **0.24** | **0.22-0.28** |
| Resumption from prior discontinuation | 1.05 | 0.99-1.11 | 0.96 | 0.88-1.05 |
| PDMP: prescription drug monitoring program, aHR: adjusted hazard ratio, 95% CI: 95% confidence intervals, MME: milligram morphine equivalents  ^a^ Baseline: 180-day period prior to study entry  ^b^ Baseline and/or follow-up: 180-day period prior to patient-month  ^c^ Low dose: <50 MME daily, High dose: ≥50 MME daily, Short-term: in 60 days, Long-term: in 180 days | | | | |

**Appendix 5: Comparison of Time-varying Cox Proportional Models for primary (20-day overlap in 30-day period) and secondary (60-day overlap in 90-day period) definitions of long-term co-prescription,** **US sample of commercially and/or Medicare Advantage insured (OLDW) cohort, 2016—2021**

|  | **20 out of 30 days with overlap**  **(n=20 425)** | | **60 out of 90 days with overlap**  **(n=10 127)** | |
| --- | --- | --- | --- | --- |
| **Independent variables** | **aHR** | **95% CI** | **aHR** | **95% CI** |
| Patient age at study entry, (Ref = 18-39) |  |  |  |  |
| 40-65 | **0.94** | **0.89-1.00** | **0.91** | **0.83-0.99** |
| More than 65 | **0.91** | **0.85-0.98** | **0.82** | **0.75-0.91** |
| Patient sex, (Ref = Female) |  |  |  |  |
| Male | **0.80** | **0.78-0.83** | **0.89** | **0.85-0.93** |
| Insurance status, (Ref = Commercial insurance) |  |  |  |  |
| Any Medicare Advantage | 0.97 | 0.93-1.00 | **1.10** | **1.04-1.16** |
| Rural-urban commuting area designation^a^, (Ref = Metropolitan) |  |  |  |  |
| Micropolitan | 1.02 | 0.98-1.07 | **1.07** | **1.01-1.14** |
| Small town or rural area | **0.94** | **0.89-0.99** | 0.99 | 0.92-1.06 |
| Median educational level^a^, (Ref = High school diploma or less) |  |  |  |  |
| Less than bachelor’s degree | **0.97** | **0.94-1.00** | **0.93** | **0.89-0.97** |
| Bachelor’s degree plus | 1.01 | 0.95-1.07 | 0.94 | 0.87-1.03 |
| Median household income^a^, (Ref = <$40,000) |  |  |  |  |
| $40,000-$74,999 | **1.07** | **1.03-1.11** | **1.07** | **1.02-1.13** |
| $75,000-$124,999 | **1.10** | **1.06-1.15** | **1.07** | **1.01-1.14** |
| $125,000-$199,999 | **1.17** | **1.10-1.25** | 1.10 | 1.00-1.21 |
| ≥$200,000 | **1.28** | **1.17-1.41** | **1.18** | **1.02-1.36** |
| Baseline^b^ average 30-day dose (MME), (Ref = 1-19) |  |  |  |  |
| 20-49 | **1.63** | **1.57-1.69** | **1.81** | **1.71-1.91** |
| 50-89 | **2.08** | **1.97-2.19** | **2.33** | **2.16-2.52** |
| 90-149 | **2.36** | **2.20-2.53** | **2.80** | **2.53-3.09** |
| 150 or more | **2.66** | **2.47-2.86** | **3.59** | **3.24-3.97** |
| Baseline^b^ total opioid prescribers, (Ref = Single Prescriber) |  |  |  |  |
| 2 prescribers | **0.90** | **0.87-0.93** | **0.87** | **0.83-0.91** |
| 3 prescribers | **0.88** | **0.84-0.92** | **0.80** | **0.75-0.86** |
| 4 or more prescribers | **0.86** | **0.81-0.92** | **0.80** | **0.73-0.88** |
| Predominant opioid type during baseline^b^, (Ref = Hydrocodone) |  |  |  |  |
| Codeine | 1.03 | 0.94-1.12 | 1.11 | 0.97-1.26 |
| Fentanyl | **0.75** | **0.69-0.82** | **0.66** | **0.58-0.74** |
| Hydromorphone | 0.92 | 0.82-1.04 | 0.87 | 0.73-1.03 |
| Methadone | **0.70** | **0.62-0.78** | **0.67** | **0.57-0.78** |
| Morphine | **0.76** | **0.71-0.83** | **0.72** | **0.65-0.81** |
| Oxycodone | **0.90** | **0.87-0.94** | **0.90** | **0.86-0.96** |
| Tramadol | **0.82** | **0.79-0.86** | **0.82** | **0.77-0.88** |
| Others (e.g., multiple opioid types) | **0.74** | **0.67-0.81** | **0.76** | **0.66-0.86** |
| Any non-fatal overdose during baseline^c^, (Ref = None) | **1.25** | **1.10-1.42** | **1.29** | **1.09-1.53** |
| Mental health status,^d^ (Ref = No diagnosis or serotonergic anxiolytic^e^ fills^f^) |  |  |  |  |
| Anxiety without SSRI/SNRI/buspirone fill | **2.16** | **2.06-2.27** | **2.32** | **2.16-2.48** |
| Anxiety with SSRI/SNRI/buspirone fill | **0.63** | **0.59-0.67** | **0.62** | **0.57-0.67** |
| Depression without SSRI/SNRI/buspirone fill | **1.16** | **1.09-1.23** | **1.22** | **1.12-1.33** |
| Depression with SSRI/SNRI/buspirone fill | **0.85** | **0.79-0.92** | **0.82** | **0.74-0.91** |
| SSRI/SNRI/buspirone fill^e^ without diagnosis | **1.51** | **1.46-1.57** | **1.57** | **1.49-1.65** |
| Alcohol use disorder | **0.89** | **0.79-1.00** | 0.94 | 0.80-1.09 |
| Substance use disorder | 1.01 | 0.96-1.06 | 1.01 | 0.95-1.08 |
| Psychoses | **1.19** | **1.13-1.25** | **1.32** | **1.23-1.42** |
| Year of cohort entry (Ref = 2016) |  |  |  |  |
| 2017 | **1.56** | **1.50-1.62** | **1.58** | **1.50-1.67** |
| 2018 | **1.55** | **1.48-1.61** | **1.61** | **1.51-1.71** |
| 2019 | **1.43** | **1.37-1.50** | **1.55** | **1.45-1.66** |
| 2020 | **1.27** | **1.20-1.34** | **1.33** | **1.22-1.44** |
| 2021 | **1.16** | **1.07-1.25** | 0.93 | 0.82-1.06 |
| Buprenorphine status, (Ref = No buprenorphine) |  |  |  |  |
| Buprenorphine initiation and no use prior 30 days | **1.64** | **1.27-2.11** | **1.68** | **1.23-2.29** |
| Buprenorphine initiation and use prior 30 days | **2.10** | **1.71-2.59** | **2.74** | **2.16-3.48** |
| Any long-acting opioid fill^f^, (Ref = No) | **0.90** | **0.86-0.95** | **0.86** | **0.80-0.93** |
| Any multiple opioid fill overlap^f^, (Ref = No) | **1.17** | **1.13-1.21** | **1.22** | **1.16-1.29** |
| Any opioid/non-benzodiazepine sedative overlap^f^, (Ref = No) | **1.05** | **1.02-1.08** | **1.08** | **1.04-1.12** |
| Any psychostimulant fill^f^, (Ref = No) | **1.34** | **1.26-1.42** | **1.37** | **1.26-1.49** |
| Short-term (60-day) dose (MME) trajectory, (Ref = Stable) |  |  |  |  |
| Decrease | **1.06** | **1.01-1.12** | **1.12** | **1.04-1.20** |
| Increase | **1.13** | **1.07-1.18** | **1.11** | **1.03-1.19** |
| Long-term (180-day) dose trajectory, (Ref = Stable) |  |  |  |  |
| Decrease | **0.78** | **0.73-0.84** | **0.86** | **0.78-0.95** |
| Increase | 0.99 | 0.95-1.03 | 1.05 | 0.99-1.10 |
| Long-term (180-day) dose variability, (Ref = Low) |  |  |  |  |
| Moderate | 0.97 | 0.94-1.01 | **0.93** | **0.89-0.98** |
| High | 0.98 | 0.94-1.03 | **0.88** | **0.82-0.94** |
| Opioid discontinuation status,^g^ (Ref = No discontinuation) |  |  |  |  |
| Low dose short-term discontinuation | 0.76 | 0.70-0.83 | **0.67** | **0.59-0.76** |
| High dose short-term discontinuation | **0.72** | **0.55-0.94** | 0.71 | 0.50-1.01 |
| Any dose long-term discontinuation | **0.30** | **0.26-0.34** | **0.27** | **0.23-0.32** |
| Resumption from prior discontinuation | 1.01 | 0.91-1.12 | 1.06 | 0.93-1.22 |
| Baseline^b^ elixhauser comorbidity indicators^d^ |  |  |  |  |
| Acquired immune deficiency syndrome | 1.05 | 0.84-1.30 | 1.29 | 0.99-1.68 |
| Deficiency anemias | 1.02 | 0.97-1.06 | 1.05 | 0.99-1.12 |
| Autoimmune conditions | 1.02 | 0.98-1.07 | 0.96 | 0.90-1.03 |
| Chronic blood loss anemia | **0.85** | **0.73-0.99** | 0.89 | 0.72-1.10 |
| Cerebrovascular disease | 1.03 | 0.96-1.10 | 1.07 | 0.98-1.18 |
| Coagulopathy | 0.97 | 0.87-1.08 | 0.96 | 0.82-1.12 |
| Dementia | **0.87** | **0.80-0.95** | **0.70** | **0.61-0.80** |
| Diabetes with chronic complications | **0.91** | **0.88-0.95** | **0.89** | **0.84-0.95** |
| Diabetes without chronic complications | 0.98 | 0.94-1.02 | 0.96 | 0.90-1.02 |
| Congestive heart failure | 1.01 | 0.95-1.07 | 1.02 | 0.93-1.11 |
| Hypertension, complicated | **0.88** | **0.82-0.94** | **0.82** | **0.75-0.90** |
| Hypertension, uncomplicated | **0.93** | **0.90-0.96** | **0.93** | **0.89-0.97** |
| Liver disease, mild | 1.03 | 0.97-1.10 | **1.10** | **1.01-1.20** |
| Liver disease, moderate to severe | 1.01 | 0.82-1.25 | 0.84 | 0.61-1.14 |
| Chronic pulmonary disease | **1.09** | **1.06-1.13** | **1.13** | **1.08-1.18** |
| Neurological disorders affecting movement | **1.14** | **1.07-1.22** | 1.07 | 0.97-1.18 |
| Other neurological disorders | 1.04 | 0.96-1.12 | **1.12** | **1.00-1.24** |
| Seizures and epilepsy | **1.13** | **1.04-1.23** | **1.19** | **1.07-1.34** |
| Obesity | **0.95** | **0.92-0.98** | **0.92** | **0.88-0.97** |
| Paralysis | 0.96 | 0.86-1.07 | 0.89 | 0.76-1.05 |
| Peripheral vascular disease | 0.96 | 0.92-1.01 | **0.92** | **0.86-0.99** |
| Pulmonary circulation disease | 0.98 | 0.87-1.09 | 1.01 | 0.85-1.18 |
| Renal failure, moderate | **0.91** | **0.86-0.97** | 0.92 | 0.84-1.00 |
| Renal failure, severe | 0.87 | 0.75-1.00 | 0.82 | 0.66-1.02 |
| Hypothyroidism | **1.07** | **1.03-1.11** | **1.07** | **1.01-1.13** |
| Other thyroid disorders | 1.08 | 1.00-1.17 | 0.94 | 0.83-1.06 |
| Peptic ulcer with bleeding | 1.01 | 0.89-1.14 | 0.91 | 0.75-1.09 |
| Valvular disease | **1.08** | **1.01-1.15** | 1.02 | 0.92-1.11 |
| Weight loss | 0.99 | 0.92-1.08 | 1.03 | 0.92-1.16 |
| OLDW: Optum Labs Data Warehouse, aHR: adjusted hazard ratio, 95% CI: 95% confidence intervals, MME: milligram morphine equivalents  ^a^ Characteristics represent patient residence census block-level measurements  ^b^ Baseline: 180-day period prior to study entry  ^c^ Baseline: 365-day period prior to study entry  ^d^ Identified from AHRQ Elixhauser comorbidity index in the 180-day period prior to study entry.  ^e^ Selective serotonin reuptake inhibitors, serotonin and norepinephrine reuptake inhibitors, and buspirone  ^f^ Baseline and/or follow-up: 180-day period prior to patient-month  ^g^ Low dose: <50 MME daily, High dose: ≥50 MME daily, Short-term: in 60 days, Long-term: in 180 days | | | | |

**Appendix 6: Observation period (30-day) characteristics within each cohort, by secondary definition (60-day overlap in 90-day period) for long-term co-prescription**

|  | **PDMP**  (N=13 817 658) | | |  | **OLDW**  (N= 5 506 314) | | |
| --- | --- | --- | --- | --- | --- | --- | --- |
|  |  | **Co-prescription Status** | |  |  | **Co-prescription Status** | |
|  | **Total** | **Yes**  (n=26 721) | **No**  (n=13 790 397) |  | **Total** | **Yes**  (n=10 127) | **No**  (n=5 496 187) |
| **Independent variables** | **%** | **%** | **%** |  | **%** | **%** | **%** |
| Patient age at study entry |  |  |  |  |  |  |  |
| 18-39 | 10.4 | 13.1 | 10.4 |  | 4.9 | 6.1 | 4.9 |
| 40-65 | 58.4 | 60.3 | 58.4 |  | 50.3 | 57.4 | 50.3 |
| >65 | 31.2 | 26.6 | 31.2 |  | 44.8 | 36.5 | 44.8 |
| Patient sex |  |  |  |  |  |  |  |
| Female | 52.8 | 60.7 | 52.8 |  | 58.4 | 63.6 | 58.4 |
| Male | 47.2 | 39.3 | 47.2 |  | 41.6 | 36.4 | 41.6 |
| Insurance status^a^ |  |  |  |  |  |  |  |
| Commercial insurance |  |  |  |  | 29.3 | 28.8 | 29.3 |
| Any Medicare Advantage |  |  |  |  | 70.7 | 71.2 | 70.7 |
| Rural-urban commuting area designation^a^ |  |  |  |  |  |  |  |
| Metropolitan |  |  |  |  | 80.3 | 78.8 | 80.3 |
| Micropolitan |  |  |  |  | 10.7 | 12.2 | 10.7 |
| Small town or rural area |  |  |  |  | 8.9 | 9.0 | 8.9 |
| Unknown |  |  |  |  | 0.1 | 0.1 | 0.1 |
| Census-block Median educational level^a^ |  |  |  |  |  |  |  |
| Less than or high school diploma |  |  |  |  | 37.7 | 39.9 | 37.7 |
| Less than bachelor’s degree |  |  |  |  | 52.7 | 50.9 | 52.7 |
| Bachelor’s degree plus |  |  |  |  | 7.9 | 7.6 | 7.9 |
| Unknown |  |  |  |  | 1.7 | 1.7 | 1.7 |
| Census-block Median household income^a^ |  |  |  |  |  |  |  |
| <$40,000 |  |  |  |  | 40.7 | 41.5 | 40.7 |
| $40,000-$74,999 |  |  |  |  | 27.8 | 27.4 | 27.8 |
| $75,000-$124,999 |  |  |  |  | 19.1 | 18.2 | 19.1 |
| $125,000-$199,999 |  |  |  |  | 5.5 | 5.4 | 5.5 |
| ≥$200,000 |  |  |  |  | 2.2 | 2.3 | 2.2 |
| Unknown |  |  |  |  | 4.7 | 5.1 | 4.7 |
| Baseline^b^ average daily dose (MME) |  |  |  |  |  |  |  |
| 1-19 | 31.0 | 20.7 | 31.0 |  | 40.0 | 23.0 | 40.0 |
| 20-49 | 38.9 | 39.2 | 38.9 |  | 35.2 | 39.5 | 35.2 |
| 50-89 | 14.5 | 17.3 | 14.5 |  | 12.1 | 17.2 | 12.1 |
| 90-149 | 7.1 | 9.9 | 7.1 |  | 5.9 | 9.0 | 5.9 |
| 150 or more | 8.5 | 12.9 | 8.5 |  | 6.8 | 11.4 | 6.8 |
| Baseline^b^ total opioid prescribers |  |  |  |  |  |  |  |
| 1 prescriber | 45.8 | 43.0 | 45.8 |  | 57.2 | 57.9 | 57.2 |
| 2 prescribers | 31.0 | 30.0 | 31.0 |  | 27.8 | 26.9 | 27.8 |
| 3 prescribers | 14.1 | 15.1 | 14.1 |  | 9.8 | 9.7 | 9.8 |
| 4 or more prescribers | 9.1 | 11.9 | 9.1 |  | 4.6 | 5.1 | 4.6 |
| Predominant opioid type during baseline^b^ |  |  |  |  |  |  |  |
| Codeine | 3.0 | 2.6 | 3.0 |  | 2.5 | 2.4 | 2.5 |
| Fentanyl | 3.0 | 4.2 | 3.0 |  | 3.1 | 3.8 | 3.1 |
| Hydrocodone | 52.0 | 49.3 | 52.0 |  | 35.9 | 35.5 | 35.9 |
| Hydromorphone | 1.1 | 1.6 | 1.1 |  | 0.9 | 1.4 | 0.9 |
| Methadone | 3.3 | 3.7 | 3.3 |  | 1.8 | 2.0 | 1.8 |
| Morphine | 6.0 | 6.2 | 6.0 |  | 4.4 | 5.2 | 4.4 |
| Oxycodone | 16.4 | 21.2 | 16.3 |  | 24.3 | 32.6 | 24.3 |
| Tramadol | 13.8 | 9.7 | 13.8 |  | 24.7 | 14.2 | 24.7 |
| Other (e.g., multiple opioid types) | 1.6 | 1.7 | 1.6 |  | 2.5 | 3.0 | 2.5 |
| Any non-fatal overdose during baseline^c^ |  |  |  |  | 0.6 | 1.4 | 0.6 |
| Mental health diagnoses^b,d^ |  |  |  |  |  |  |  |
| Anxiety |  |  |  |  | 15.7 | 32.7 | 15.7 |
| Alcohol use disorder |  |  |  |  | 1.3 | 1.7 | 1.3 |
| Depression |  |  |  |  | 16.5 | 25.5 | 16.5 |
| Substance use disorder |  |  |  |  | 7.8 | 10.6 | 7.8 |
| Psychoses |  |  |  |  | 4.6 | 9.3 | 4.6 |
| Buprenorphine status |  |  |  |  |  |  |  |
| No buprenorphine | 99.2 | 99.1 | 99.2 |  | 99.2 | 98.9 | 99.2 |
| Buprenorphine initiation and no use prior 30 days | 0.4 | 0.4 | 0.4 |  | 0.4 | 0.4 | 0.4 |
| Buprenorphine initiation and use prior 30 days | 0.4 | 0.6 | 0.4 |  | 0.4 | 0.7 | 0.4 |
| Any long-acting opioid fill^f^ | 20.4 | 28.5 | 20.4 |  | 16.0 | 23.3 | 16.0 |
| Any multiple opioid fill overlap^f^ | 26.9 | 42.2 | 26.8 |  | 20.7 | 31.2 | 20.7 |
| Any opioid-Z-drug overlap^f^ | 13.6 | 28.6 | 13.6 |  | 37.5 | 47.5 | 37.5 |
| Any stimulant fill^f^ | 3.5 | 6.2 | 3.5 |  | 3.0 | 5.9 | 3.0 |
| Any serotonergic anxiolytic^e^ fill^f^ |  |  |  |  | 34.8 | 48.8 | 34.8 |
| Short-term (60-day) dose (MME) trajectory |  |  |  |  |  |  |  |
| Decrease | 9.2 | 12.2 | 9.2 |  | 7.6 | 9.7 | 7.6 |
| Stable | 81.3 | 74.2 | 81.3 |  | 84.8 | 80.2 | 84.8 |
| Increase | 9.5 | 13.6 | 9.5 |  | 7.6 | 10.1 | 7.6 |
| Long-term (180-day) dose trajectory |  |  |  |  |  |  |  |
| Decrease | 9.5 | 7.8 | 9.5 |  | 7.8 | 6.2 | 7.8 |
| Stable | 70.8 | 65.4 | 70.8 |  | 73.2 | 71.4 | 73.2 |
| Increase | 19.7 | 26.9 | 19.7 |  | 19.0 | 22.4 | 19.0 |
| Long-term (180-day) dose variability |  |  |  |  |  |  |  |
| Low | 41.0 | 32.3 | 41.0 |  | 48.0 | 42.7 | 48.0 |
| Moderate | 34.1 | 37.3 | 34.1 |  | 30.9 | 35.1 | 30.9 |
| High | 24.9 | 30.4 | 24.9 |  | 21.2 | 22.2 | 21.2 |
| Opioid discontinuation status^g^ |  |  |  |  |  |  |  |
| No discontinuation | 83.6 | 94.6 | 83.6 |  | 77.5 | 92.6 | 77.5 |
| Low dose short-term discontinuation | 5.4 | 2.0 | 5.5 |  | 8.6 | 3.3 | 8.6 |
| High dose short-term discontinuation | 0.5 | 0.4 | 0.5 |  | 0.4 | 0.3 | 0.4 |
| Any dose long-term discontinuation | 7.3 | 0.9 | 7.3 |  | 9.3 | 1.4 | 9.3 |
| Resumption from prior discontinuation | 3.1 | 2.1 | 3.1 |  | 4.3 | 2.4 | 4.3 |
| **Note:** Empty cells indicate variables not available for analysis in that cohort  PDMP: California prescription drug monitoring program data, OLDW: Optum Labs Data Warehouse, MME: Milligram morphine equivalents  ^a^ Variables measured at census block level  ^b^ Baseline: 180-day period prior to study entry  ^c^ Baseline: 365-day period prior to study entry  ^d^ Identified from AHRQ Elixhauser comorbidity index  ^e^ Selective serotonin reuptake inhibitors, serotonin and norepinephrine reuptake inhibitors, and buspirone  ^f^  Baseline and/or follow-up: 180-day period prior to patient-month  ^g^ Low dose: <50 MME daily, High dose: ≥50 MME daily, Short-term: in 60 days, Long-term: in 180 days | | | | | | | |

**Supplemental References**

1. Vivolo-Kantor A, Pasalic E, Liu S, Martinez PD, Gladden RM, Overdose Morbidity T. Defining indicators for drug overdose emergency department visits and hospitalisations in ICD-10-CM coded discharge data. Inj Prev. 2021 Mar;27(S1):i56-i61.

2. Moore BJ, White S, Washington R, Coenen N, Elixhauser A. Identifying Increased Risk of Readmission and In-hospital Mortality Using Hospital Administrative Data: The AHRQ Elixhauser Comorbidity Index. Med Care. 2017 Jul;55(7):698-705.
